# Endoscopic management of gastric band erosion using argon plasma coagulation

**DOI:** 10.1016/j.vgie.2025.01.001

**Published:** 2025-01-07

**Authors:** Emmanuel Palomera-Tejeda, Kalpesh Patel, Farid Abushamat, Salmaan Jawaid

**Affiliations:** Division of Gastroenterology, Baylor College of Medicine, Houston, Texas, USA

Gastric banding emerged as one of the first bariatric procedures; however, its use has significantly decreased in favor of more modern interventions.^1^, ^2^, ^3^ Gastric banding accounted for less than 1% of total bariatric interventions in the United States in 2019, compared with 35.4% in 2011.^1^ This decline is primarily the result of limited weight loss and a high rate of adverse events, such as gastric band erosion (GBE). GBE is a rare but well-known and dangerous adverse event in which the band erodes into the stomach and may further migrate, causing biliary or bowel obstruction.^2^ The management of GBE involves band removal, which can be performed using an endoscopic and/or surgical approach. However, an initial endoscopic approach is preferred to monitor for adverse events in real time, as the external aspect of the band usually is encapsulated in fibrosis, and a laparoscopic approach carries a risk of inadvertent gastric perforation.^3^, ^4^, ^5^

## Aim

The objective of this video case report is to demonstrate the use of argon plasma coagulation (APC) to facilitate the endoscopic removal of an eroded gastric band.

## Case report

A 68-year-old woman presented for evaluation of gastric band removal. She had the band placed approximately 30 years previously, with an initial rapid loss of 70 lbs reported. Over time, she experienced chronic abdominal discomfort and intermittent nausea and vomiting, with recent worsening symptoms. Previous investigations included an EGD with a reported eroded band. She was referred to our medical center for further management and was scheduled for an index EGD with possible band removal.

## Procedure

**Figure 1:**
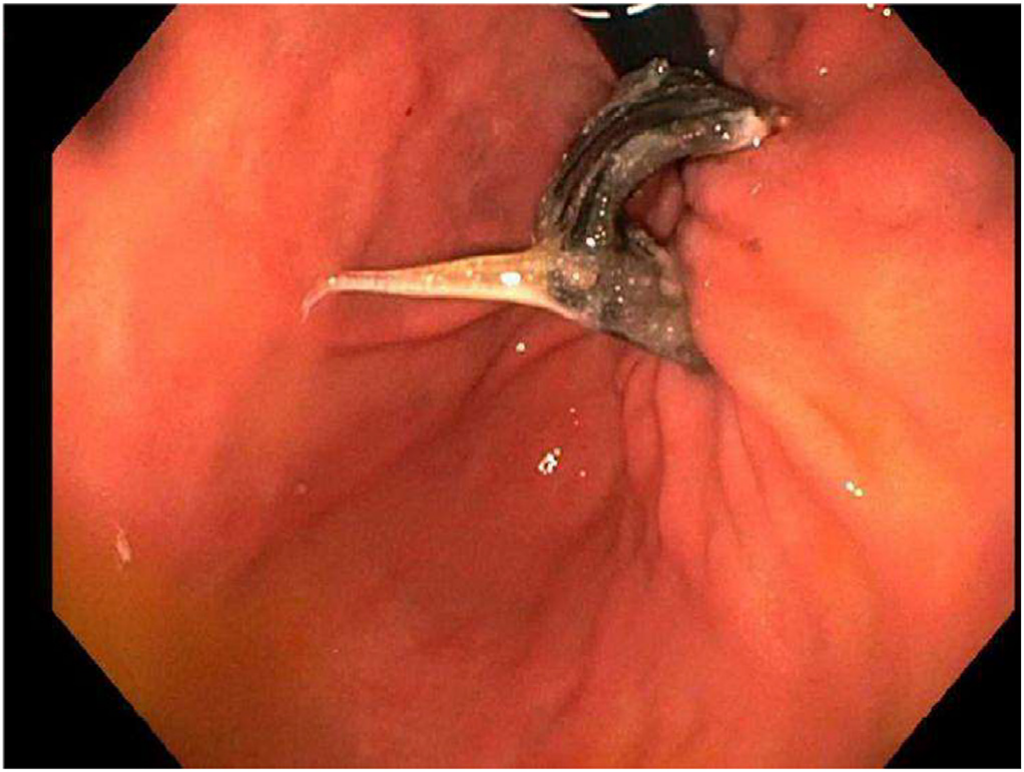
Migrated gastric band with 75% of its circumference eroding into the stomach fundus.

**Figure 2:**
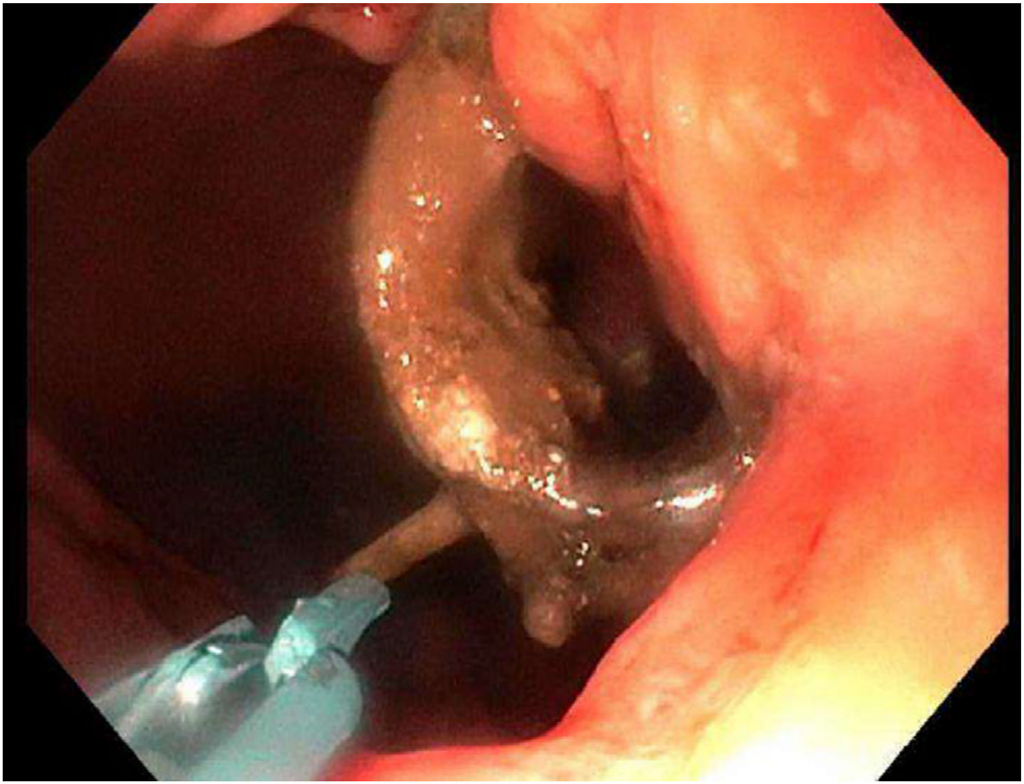
Initial transection attempt using an endoscopic knife.

**Figure 3:**
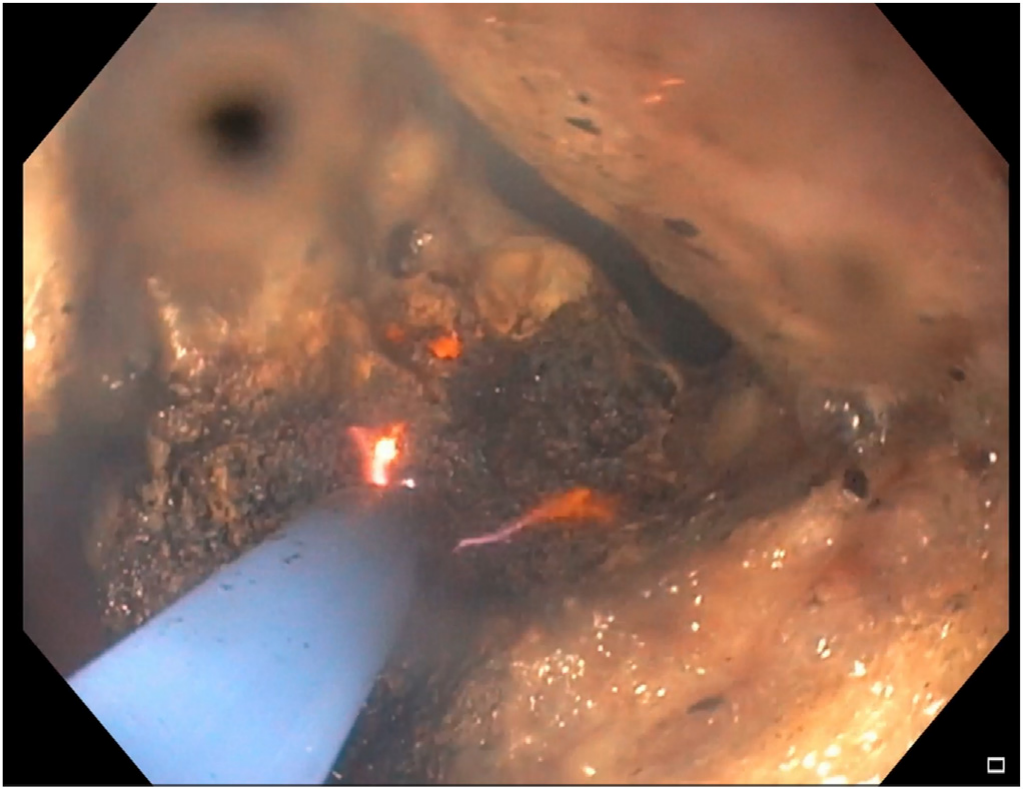
Application of argon plasma coagulation to facilitate the transection of the band.

**Figure 4:**
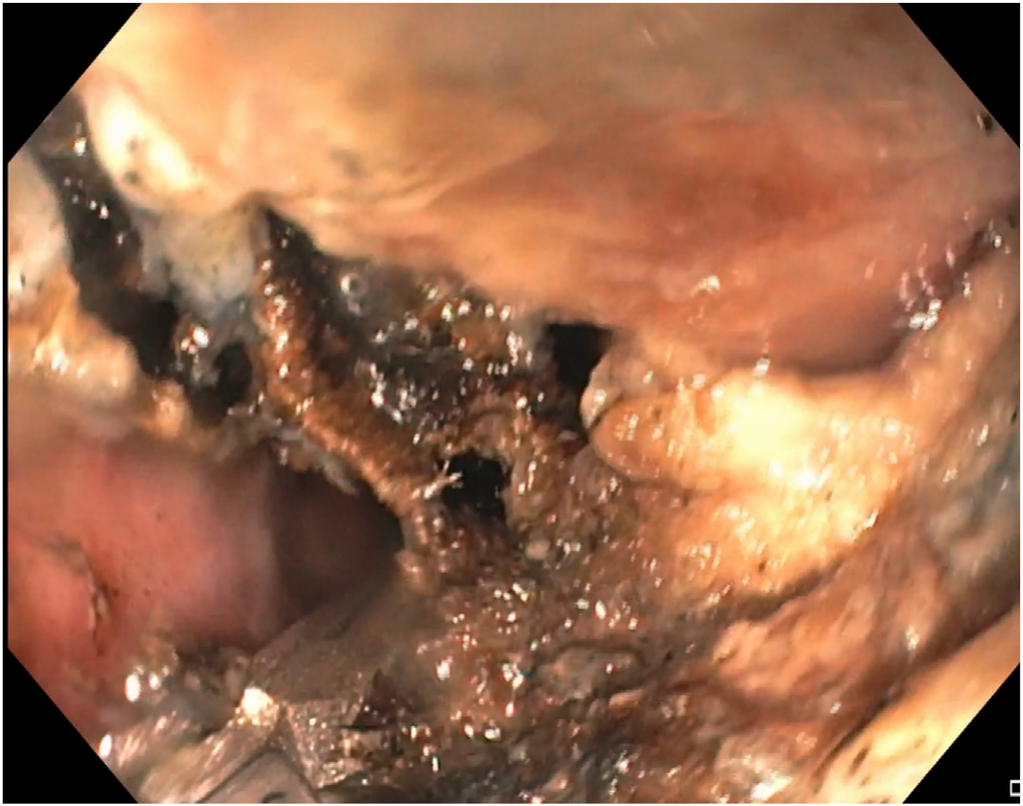
Final separation from the gastric wall using a Raptor grasping device.

**Figure 5:**
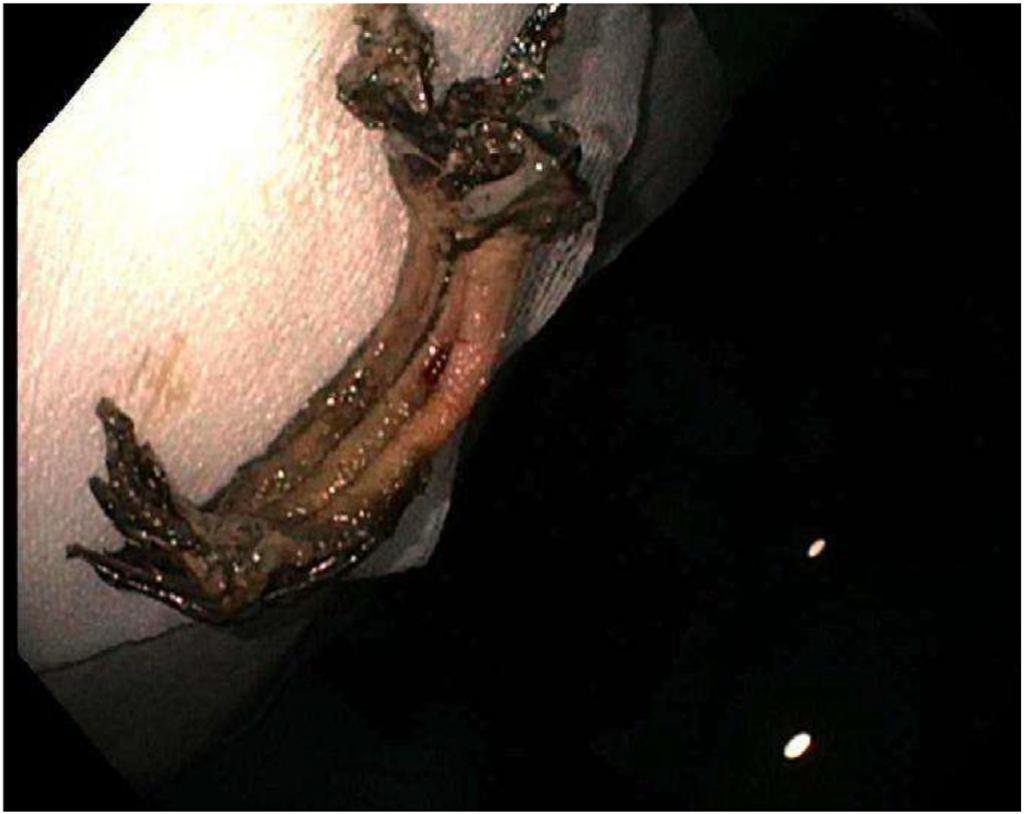
Transected gastric band after retrieval.

During the EGD (GIF-HQ190; Olympus Corp, Center Valley, Pa, USA), an eroded lap band, 75% exposed, was found in the gastric fundus (Fig. 1 and Video 1, available online at www.videogie.org). An endoscopic knife (SB knife; Olympus Corp) was used as an initial attempt to sever the band, but progress was slow (Fig. 2). To minimize tissue manipulation of the already damaged gastric mucosa, forced APC at 100 W using straight-fire axial beam (VIO 3; Erbe USA, Marietta, Ga, USA) was applied to the band to facilitate transection (Fig. 3), with a total APC duration of 3.5 minutes. Complete separation from the gastric wall and retrieval of the transected band (Figs. 4 and 5) were successfully accomplished with a foreign body–grasping forceps (Raptor; Steris, Mentor, Ohio, USA).

## Outcome

**Figure 6:**
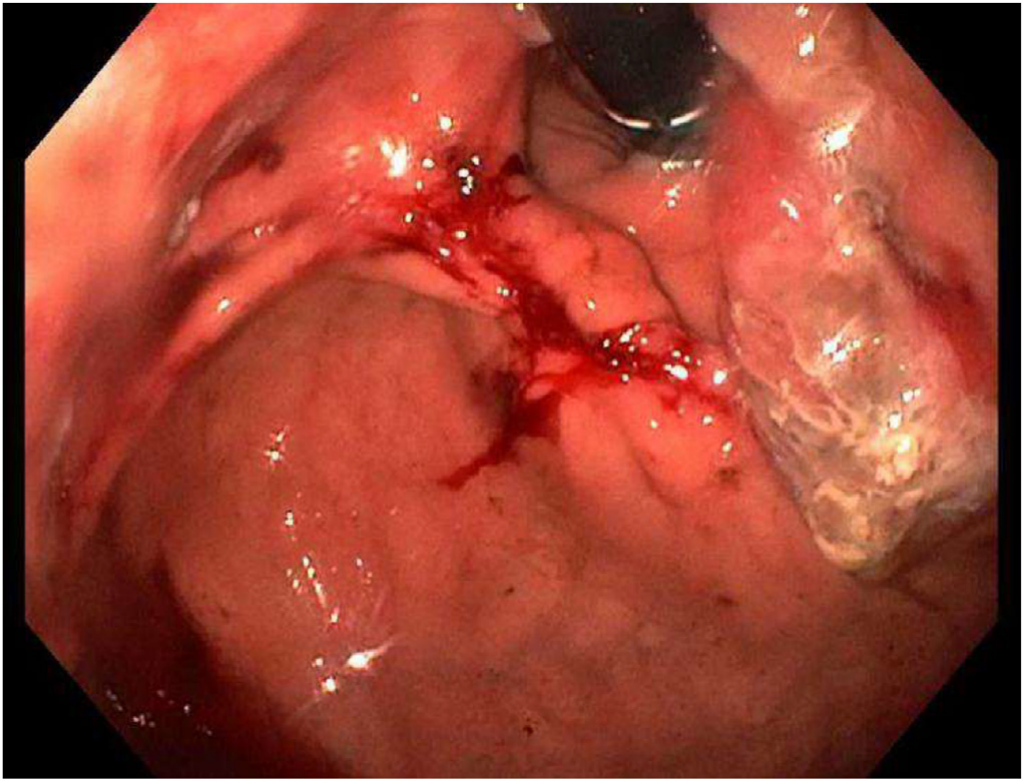
Gastric fundus after endoscopic band removal.

The patient's course was satisfactory (Fig. 6), and she was discharged on the same day as the procedure. She continued follow-up with the bariatric service for further management of the subcutaneous port, and her symptoms had already improved by her next visit.

## Conclusions

In conclusion, gastric band migration is a rare but still-encountered adverse event of gastric banding. Prompt removal of the eroded band is essential to prevent further adverse events. Endoscopic removal can be achieved using various endoscopic tools; however, it can be quite challenging because of the resistance of the band. In this video case report, we demonstrate the feasibility of using APC to facilitate the endoscopic transection of an eroded band and potentially reduce the risk of intraprocedural adverse events. Although APC is a useful adjunctive tool that is easy to operate and widely available, its success is not guaranteed and may necessitate additional removal techniques. Effective alternatives include endoscopic scissors and mechanical lithotripters with guidewire assistance.^3^^,^^5^

## Patient Consent

The patient in this article has given informed consent to publication of the case details.

## Disclosure

All authors disclosed no financial relationships.

## Supplementary data

Video 1 Management of gastric band erosion using argon plasma coagulation.
